# Exploring Perceptions of Colorectal Cancer and Fecal Immunochemical Testing Among African Americans in a North Carolina Community

**Published:** 2011-10-15

**Authors:** Elizabeth Harden, Alexis Moore, Cathy Melvin

**Affiliations:** University of North Carolina at Chapel Hill, Chapel Hill, North Carolina; Lineberger Comprehensive Cancer Center, University of North Carolina at Chapel Hill; University of North Carolina at Chapel Hill, Chapel Hill, North Carolina

## Abstract

**Introduction:**

African Americans have a lower colorectal cancer screening rate than whites and higher disease incidence and mortality. Despite wide acceptance of colonoscopy for accurate screening, increasing promotion of high-sensitivity stool test screening, such as the fecal immunochemical test (FIT), may narrow racial, ethnic, and socioeconomic disparities in screening. This study provides formative research data to develop an intervention to increase colorectal cancer screening among underinsured and uninsured African Americans in central North Carolina.

**Methods:**

We held 4 focus groups to explore knowledge, beliefs, and attitudes about colorectal cancer screening, particularly FIT. Participants (n = 28) were African American adults recruited from neighborhoods with high levels of poverty and unemployment. Constructs from the diffusion of innovation theory were used to develop the discussion guide.

**Results:**

In all groups, participants noted that lack of knowledge about colorectal cancer contributes to low screening use. Attitudes about FIT sorted into 4 categories of "innovation characteristics": *relative advantage* of FIT compared with no screening and with other screening tests; *compatibility* with personal beliefs and values; test *complexity*; and test *trialability.* A perceived barrier to FIT and other stool tests was risk of incurring costs for diagnostic follow-up.

**Conclusion:**

Community-based FIT screening interventions should include provider recommendation, patient education to correctly perform FIT, modified FIT design to address negative attitudes about stool tests, and assurance of affordable follow-up for positive FIT results.

## Introduction

Screening for colorectal cancer (CRC), the second leading cause of cancer death in the United States, leads to increased early detection and treatment of this disease (1). Although 62% of the US population reports following recommended CRC screening guidelines, screening rates are lower for those with lower incomes and those without health insurance (2). Rates are also thought to be lower among African Americans, who are more likely than other ethnic groups to be diagnosed with late-stage CRC (2) and 40% more likely than whites to die of CRC (3).

Adults at average risk for CRC may choose from several screening options. Until recently, organizations that develop and issue guidelines were generally in consensus about which tests to endorse. The US Preventive Services Task Force (USPSTF) recommends that patients choose from among yearly high-sensitivity fecal occult blood test (FOBT) or high-sensitivity fecal immunochemical test (FIT), flexible sigmoidoscopy every 5 years, or colonoscopy every 10 years. Each screening regimen is clinically effective for reducing mortality (4,5).

In 2008, the American Cancer Society (ACS), in collaboration with the US Multi-Society Task Force on Colorectal Cancer and the American College of Radiology, issued a slightly diverging set of recommendations, adding stool DNA and computed tomographic colonoscopy tests. The ACS guidelines also categorize tests according to their potential to detect versus prevent CRC. The first category includes FOBT and FIT. The second category includes screening tests that produce visual images of the colon and, therefore, can detect and guide removal of adenomatous polyps, a benign precursor of most colorectal cancers, thereby preventing them from developing into cancer (6).

With either set of guidelines, patients must choose a test that most closely aligns with their needs and values. Each test conveys distinct benefits and limitations related to test frequency, cost, invasiveness, sensitivity, specificity, convenience, and regional availability. For lay and professional audiences alike, determining the relative advantage of the various screening tests is a complex issue.

FOBT and FIT are the least expensive options and do not require access to endoscopy facilities. Some types of FIT are increasingly preferred over FOBT because the tests are specific to human hemoglobin and have a similarly high or higher sensitivity (7). Vitamins, foods, and drugs do not alter FIT accuracy, and patients may find it easier to use. Improving access to FIT has potential to increase screening by reducing costs and removing some structural barriers, such as geographically distant endoscopy facilities (8-12).

The objective of this study was to provide formative research data for an intervention to increase CRC screening in a target population of underinsured and uninsured African Americans living in a metropolitan area in central North Carolina. We conducted focus groups with members of the target population to explore knowledge, beliefs, and attitudes about CRC screening, particularly FIT. Focus group data are being used to inform the design of a FIT screening intervention.

## Methods

### Theoretical framework: diffusion of innovations

The diffusion of innovations theory describes the adoption of new practices or products (innovations) and the factors that accelerate or impede their spread throughout a community. Application of this theory during intervention planning can help cancer prevention and control practitioners develop dissemination strategies specific to different CRC screening tests and populations.

The theory posits that perceptions of an innovation's characteristics affect how quickly and widely the innovation is adopted. Five attributes that explain 49% to 87% of variance in adoption rates are *relative advantage*, *compatibility*, *complexity*, *trialability*, and *observability* (13). Relative advantage is the degree to which a potential user perceives the innovation as superior to the practice that it supersedes. Compatibility refers to the beliefs about whether the innovation is consistent with personal values. Complexity is the extent to which the user perceives the innovation as difficult to use. Trialability is the degree to which someone can experiment with the innovation before adopting it. Potential users can also conduct a vicarious trial by observing and learning from someone else's experimentation (13). Observability is the extent to which results of adopting an innovation are visible to others. Modifying FIT in ways that affect perceptions of these 5 attributes in the target population can enhance or diminish its diffusion potential. Intervention planning also requires audience and community assessment research to understand how an innovation is likely to interact with individual and environmental characteristics. Elements in the diffusion of innovation theory guided the organization and presentation of the focus group results.

### Procedures

Starting in January 2007, a local community research advisory board, a standing group that advises about research projects in several North Carolina counties, reviewed the research protocol, recommended appropriate honoraria for participants, and guided the research team in disseminating results locally. The institutional review board of the University of North Carolina at Chapel Hill also approved this study. Community organizations assisted in early spring 2007 by posting flyers and allowing study staff to attend their outreach events to recruit and enroll participants. Potential participants contacted research staff in person at these events or by telephone after seeing recruitment materials. Two study staff administered brief eligibility surveys and enrolled participants.

Eligible participants were African Americans aged 50 years or older who were not at elevated risk for CRC because of a family history of CRC in a first-degree relative or a personal history of the disease. Those screened by FOBT or FIT within the past year or by any endoscopy method or contrast barium enema within the past 5 years were excluded. Of 51 people who completed eligibility screening, 16 were ineligible because of age or recent screening. Thirty-five eligible people received assignment to a male or female focus group session (there were 2 of each), a confirmation letter, and a reminder letter and telephone call. Of those, 28 attended 1 of 4 two-hour focus groups and completed a brief demographic questionnaire in March 2007. Focus groups of 5 to 9 people were conducted at 2 African American churches of different denominations and at a community resource center. The churches and the resource center were recommended as neutral sites that were likely to be familiar and geographically accessible to participants. Eligible participants chose to attend either a weekend morning or weekday evening focus group. The study covered taxi costs for participants without transportation. All participants received a $30 gift certificate.

Focus group members gave written consent for their participation. A trained African American facilitator of the same sex as participants moderated the focus groups. All groups were tape recorded. Facilitators followed a semistructured guide with preset probes (Appendix) to ensure conversation depth. The moderator began with questions about participants' knowledge and attitudes about CRC and screening. After distributing a 3-sample Hemoccult ICT packet, an FIT manufactured and donated by Beckman Coulter, Inc (Brea, CA), the moderator asked participants to examine the packet and share opinions about its design, packaging, instructions, and usability. The moderator also asked participants questions about community characteristics and local health services, and participants were able to ask questions or raise topics that they thought were important but had not been addressed.

### Analysis

Verbatim transcription of audio recordings produced 191 pages of text. The first author used Atlas.ti software (Atlas.ti Scientific Software Development GmbH, Berlin, Germany) to conduct a content analysis followed by thematic analysis. Content analysis examined the degree of consensus in responses to questions and generated a list of codes that defined overarching themes. The analyst coded and ranked each comment from most to least frequently mentioned. A second analyst also reviewed transcripts, and differences in the analyses were resolved.

Thematic analysis entailed grouping codes by themes, which were defined by elements of diffusion of innovation theory. Qualifying findings had to emerge in at least 2 groups. A finding's strength increased if it occurred in 3 or all 4 groups. To count as a finding for women, a code had to occur in both women's groups, and likewise for the men. During thematic analysis, the analyst abstracted quotes that illustrated findings.

## Results

Half of study participants reported an annual income of less than $10,000 (Table). Twenty participants reported having a regular health care provider, yet only 13 had ever spoken with a provider about CRC. More women than men reported talking with a health care provider about CRC.

Themes are presented in terms of individual, innovation, and environmental characteristics that, following diffusion of innovation theory, are likely to influence FIT adoption. Comments about FIT aligned with 4 of the 5 innovation characteristics believed to predict adoption: *relative advantage* of FIT compared to no screening or other CRC screening, *compatibility* of FIT with personal beliefs and preferences, *complexity* of the FIT procedures, and strategies to enhance FIT's *trialability*. Participant comments did not directly address the innovation's *observability*; however, the low profile of CRC screening emerged as a related theme.

### Individual characteristics: awareness and knowledge of CRC and CRC screening

Across all groups, awareness and knowledge of CRC and screening were low. Female participants said that CRC screening is discussed less frequently than breast or cervical cancer screening.

You know, you do tell people "I went for a mammogram" because one of the things that women do discuss when they go for a mammogram is . . . how the test felt, you know, what was done. We talk about Pap[anicolaou] smears. But I just never, ever heard anybody say anything when they go for their physical about [CRC screening]. (Woman, group C)

### Innovation characteristics: perceptions about FIT screening

**Relative advantage**

Most participants noted that finding cancer early is beneficial relative to late diagnoses, and most indicated that FIT screening was preferable to other CRC screening tests. Several men said that they liked the idea of a home test for CRC.

There was a time I was, uh, the doctors wanted to take that [colonoscopy] for me and I wouldn't allow them because that's a part of my body. I just, just can't see nobody doing what they do. So, I told them "Ain't there no other way you can test it?" (Man, group A)

When differences between FOBT and FIT were discussed, FIT was preferred because it requires no food restrictions.

**Compatibility**

Negative attitudes about FIT were due mostly to perceptions of the test as "gross." Women expressed more reluctance about collecting and storing stool samples than men and noted that people may be deterred by smelly odors or embarrassment when returning the samples. All groups discussed problems associated with storing stool samples. Some recommended adding a device to hold used sample cards until they go to the lab.

No one thinks to let it dry completely overnight. So, I guess you have to put this someplace where — I don't know — You know, because you got to let it dry completely, and you don't want to just leave it on the sink. (Man, group D)

Participants also said stool tests are a good screening option for those preferring home remedies to medical services.

Participants generally approved of the appearance of FIT packaging; however, they thought that the thin, lightweight materials could easily be overlooked or discarded.

If I don't really have an understanding of how important it is, I'll just discard it, you know, because it looks like another piece of mail. So, it just goes in the garbage can with all my other mail. (Man, group D)

**Complexity**

Although most said FIT is a simple procedure, in every group participants said FIT's multistep instructions would challenge some. Instructions in small type were acknowledged as a potential problem for low literacy patients. Not understanding the rationale for each step of the FIT procedure, which is performed over several days, bothered some participants: "Okay, you're gonna put 2 [pieces of stool] on it, 1 for the bottom, 1 for the top. Then you're going to smear [them] together. For what?" (woman, group B). Another participant said, "All the processes . . . Lift the toilet seat. Attach it to fit you. Measuring tissue. . . . You know, it's a lot to do. A lot to do for me" (man, group A).

Others shared similar concerns.

I think before they give a person a kit like that, they just educate them on why they're doing it and what they should do, what they're going to be looking for. 'Cause if they just give it and tell them to take it home, bring their stool sample back, I mean they haven't told them nothing. (Woman, group B)

**Trialability**

A few had previously tried and completed a 3-sample FOBT. One man explained that "there's nothing to it" (group D). Another participant described how a female health care provider helped him practice a test procedure before he attempted it independently.

They went through the whole thing with me. Cause I didn't know exactly how to do it. Cause I can read and I can guess. . . . She said, "I'm gonna sit here and we'll go through the whole process." Then you know exactly what to do. (Man, group D)

In addition to hands-on instruction, video or illustrations were suggested by the participants to improve adherence to test procedures.

**Observability**

The silence surrounding CRC was discussed in all groups. One woman (group C) compared the invisibility of CRC screening with other screenings: "We hear a lot about mammograms . . . we hear a lot about cervical cancer . . . and the importance of Pap[anicolaou] smears. But we don't really hear a lot about colon cancer." Although benefits of early detection or prevention of cancer are not easily observed, negative consequences of late-stage cancer diagnosis are, prompting such remarks as "a lot of people [are] afraid of taking the necessary steps as far as being examined. I guess they're scared of what they will find out" (woman, group C).

### Environmental characteristics with potential to affect FIT screening

**Provider recommendation**

Lack of provider recommendation appeared as a screening impediment: "If the doctor tells me this [FIT] is something I must do, or I have to do it, I just have to humble myself and get it done. But until that time, man, I'll just take my personal beliefs and use them" (man, group A). Participants described physicians as important health information sources, yet rarely discussed CRC with a doctor.

**Health care cost and access**

Even if free CRC screening were available, participants said they might opt out unless affordable follow-up is assured. In the absence of diagnostic care and treatment, screening may be pointless: "The cost factor. If I do [have cancer], I can't afford to continue with the treatment or whatever is needed to be done. Therefore, if I don't start and I don't know, I won't have to follow through" (man, group A).

## Discussion

The National Commission on Prevention Priorities ranked CRC screening as the fourth most valuable clinical preventive service that medical practices can offer (14). Although some tests, particularly colonoscopy, have ardent supporters, most people agree that USPSTF- or ACS-recommended tests increase early detection of CRC (1,15) and that patients should be able to choose a test they prefer. Results from published studies indicate that patients do not unanimously favor one test over another; some studies indicate FOBT and FIT are least preferred (16,17). Others show some patients preferring stool tests over colonoscopy (9,18). Findings from our study suggest that FIT is perceived as an acceptable or preferred CRC screening test relative to other screening tests, including colonoscopy. Fisher et al (12) report that using annual stool tests for primary screening would allow 100% of the age-appropriate US population to be screened at a savings of nearly $10 billion per decade from what is currently spent to screen only half the targeted population. For cancer prevention and control planners working to extend CRC screening to underinsured and uninsured patients, our findings suggest FIT is viable for community screening. Participant comments exposed factors that could impede widespread adoption and should be taken into consideration when planning screening programs that include FIT.

Complexity of test procedures emerged as a concern. For innovations requiring acquisition of new skills, diffusion tends to be slow (13). FIT screening entails following multistep instructions over a span of several days. Other studies have found that people often lack skills and confidence to successfully complete stool tests (11,19,20). Another focus group study reported that clearer instructions about test procedures would improve participation rates (11). Similarly, participants in our study recommended adding instructions in large type and illustrations of test procedures.

Participants also recommended hands-on practice sessions using sample materials or video demonstrations. In addition to reducing complexity, these activities address the concept of trialability by allowing patients to try FIT before committing to using it at home. Three-sample FIT and FOBT are already designed with a certain degree of trialability: patients can gain confidence in doing the test as they attempt to collect a sample each day. Although test accuracy decreases with an incomplete sampling, 1 or 2 samples can still be analyzed. The next year, the patient will have another opportunity to perform the test.

Our focus group participants indicated that handling or mailing stool samples is embarrassing and mildly offensive, hence incompatible activities. Similar attitudes have been reported about FOBT (20-22). In an Australian study of FOBT use, the 2 main reasons, together accounting for almost 50% of reasons for nonadherence, were perceived unpleasantness and inconvenience (22). Participants in our study noted that merely adding a storage device to securely contain fecal samples until they are returned to the doctor may make FIT more acceptable.

In addition to the innovation characteristics of FIT screening, the public's low awareness of stool testing may impede adoption (11,19,20,23,24). The effectiveness of mass media interventions for increasing CRC screening deserves further research (25); however, the most important source of information about CRC screening is health care professionals (23,26). Although physician recommendation for screening is a leading predictor of screening adherence (23,26), only 13 of 28 participants in this study had talked with a health care provider about CRC. Physicians have reported not recommending CRC screening to uninsured patients if access to diagnostic care is lacking (11). Participants noted that FIT screening has little value unless diagnostic follow-up services are available and affordable. In regions where CRC screening programs do not fund screening and diagnostic colonoscopy for the underinsured and uninsured, adhering to CRC screening guidelines is a challenge.

Focus group data, while offering great depth and detail in response to research questions, cannot be generalized beyond the sample. In our study, convenience sampling and the small sample size further decreased generalizability of the results. Also, only 1 researcher coded the content analysis, potentially decreasing the validity of our findings.

CRC disproportionately affects the lives of African Americans, and screening rates must be increased to reduce the number of African American lives lost to the disease. Findings from our study and others indicate that FIT is a viable option for more widespread population-based CRC screening.

## Figures and Tables

**Table.** Characteristics of Participants in a 2007 Focus Group Study Conducted With African Americans (n = 28) in Central North Carolina

**Characteristic**	Women	Men	Women and Men Combined
No. of participants	14	14	28
Average age, y	65	57	61
Annual income <$10,000^a^	7	7	14
Annual income $10,000-$19,000^a^	3	4	7
Has a regular health provider	12	8	20
Has talked to provider about colorectal cancer	9	4	13

a Data on income were missing for 1 woman and 2 men.

## Appendix

**Focus Group Moderator's Guide** (DOC 51k). This file is available for download as a Microsoft Word document.

**Description: **Microsoft Word is a word processing program used to create and edit text documents. Text in Word documents can be easily modified or copied for use in other applications. **File extensions:** .doc, .rtf **Viewing: **If you do not already have Word, you can download Word Viewer for free.
