# Supplementary material for: Exploring Perceptions of Colorectal Cancer and Fecal Immunochemical Testing Among African Americans in a North Carolina Community
Source: Prev Chronic Dis. 2011 Oct 15;8(6):A134. (PMC3221575)
Supplement: Supplementary file 1 [file 10_0234_01.doc]

**Appendix**

# Focus Group Moderator's Guide

**GREETING:**

Thank you for being here today. We really appreciate you taking the time to be here with us and for your participation in this discussion.

- (Facilitator): My name is _________.
- (Note taker): My name is _________, and we’re working with the University of North Carolina at Chapel Hill.

**PURPOSE:**

**15 min** We are part of a team of researchers from University of North Carolina at Chapel Hill who are conducting a research study to find out how African American men and women feel about a type of screening test that can help detect colorectal cancer early, before the disease has a chance to spread. Colorectal cancer is cancer of the intestines. We are interested in learning more about what people think about a particular test used to screen for colorectal cancer. Your ideas, opinions, and concerns are very important to us, and they will help us to design better programs for helping people in the High Point area get screened for cancer if they choose to.

Before we begin, we’d like to stress that our research team will keep everything said here today confidential. What you say will be used only to help us design a better program. Nothing you say will be connected with your name. We ask that you too keep what you hear confidential and not repeat what we have discussed to anyone outside of this group. We hope you will feel free to speak openly but be aware of our limits in protecting your confidentiality.

We have a lot to cover, so I may need to change the subject or move ahead with the discussion. But, please stop me if you want to add anything or if you have any questions. Our discussion today will last about two hours.

**ROLES:**

(Facilitator)

My role today will be to ask some specific questions and to keep the conversation going.

(Note taker)

My job today is to take notes, but I may ask a few questions as well. It is often difficult for me to write as fast as people talk, so we would like to tape-record this discussion also. We want to be sure to get all of the important things that you say.

(Facilitator)

If at any time you would like us to stop tape recording, just ask, and we will do so. The tapes will only be heard by staff working on the project. We are now going to turn on the tape recorder. Does anyone have a problem with our tape recording the session now?

Let's begin by introducing ourselves and just to break the ice, also say one thing you do to keep yourself healthy.

Thank you for sharing that information about yourselves. Now, I’d like to ask you some specific questions, and remember there is no right or wrong answer.

**QUESTIONS:**

**PART 1. Knowledge, Attitudes, Beliefs about Screening**

**15 min** 1. Cancer screenings are one way to detect cancer early so it is more likely to be treated successfully. Today, we are going to talk about screening for one kind of cancer: colorectal cancer. What have you heard about colorectal cancer from people you know, doctors, or the media?

- Probe for thoughts about other tests (colonoscopy and sigmoidoscopy). ***If participants have questions about what any of the tests consist of, the moderator should use the attached handout to explain.***

2. What types of things might keep someone from getting colorectal cancer screening? Or what types of things would make it hard to get the screening?

**PART 2. Knowledge, Attitudes, Beliefs about FOBT/FIT**

**20 min** Looking for hidden blood in the stool (or excrement) is one way to detect colorectal cancer. This blood cannot be seen with the naked eye. There are two main types of home tests that can be used to look for blood in the stool. They are called the Fecal Immunochemical Test or “FIT” and the Fecal Occult Blood Test or “FOBT.” These tests are very similar to each other. When a patient’s doctor or nurse recommends FIT or FOBT, there are certain things a patient has to do to be sure the test is done correctly. This test is usually done at home. You collect a sample from three different stools, usually three days in a row. With FIT, you will use a stick that they give you to apply a thin smear from two different parts of the stool, and then allow the card to dry. Usually you collect the samples for three different stools, and then return all of them to your doctor at the same time, by mailing them. A test like this is usually only given to people 50 and older. And they do it once a year.

3. What words have you heard used to refer to this test?

- Probe for words *you* have used to refer to this test
- Probe for what words people feel comfortable using to refer to bowel movements
- Probe for what words people feel comfortable using to refer to rectum

4. How do you feel about this test for blood in the stool? If you’ve done this test before, please speak from your own experiences, if possible.

- Probe for likes and dislikes
- Probe for social/environmental factors
- What about the fact that **you** are responsible for doing this test — at home and on your own time — instead of having it taken care of during a doctor visit?
- Probe for familiarity with the test
- Has anyone done one before?

**PART 3. Attitudes about the FIT kit**

**1 hr** Now I’m going to pass around an FIT kit for you to look at. (pass around kits) Please take a look at your kit. I’m going to give you a few minutes to look at the information and then ask you for your initial reactions to it. If you don’t get through it all, let us know. That is useful information that we can use to make the information easier to understand. After the focus group, if you want more time to read through all of the information, you can do it then.

5. What are your initial reactions to this test?

- Probe for readability, clarity, understanding the diagrams/pictures, etc.
- Probe for packaging likes and dislikes (readability, clarity, colors)

Now, Think about your 2 best female/male friends, What would their reactions be to this test?

6. If your doctor or nurse gave you this home test, what more would you need to see and know to help you do it?

7. What might keep you from doing this test at home?

- Probe for packaging issues (e.g., easy/difficult to use, complexity related to steps)
- Probe for social and environmental factors
- Probe for embarrassment, unpleasantness, worries about discomfort

8. This test has several steps. For example, you have to collect the “stool” samples, and THEN you have to mail or take the test card to the doctor’s office or lab. What might keep you from returning the cards to your doctor or nurse?

9. What things could your doctor, nurse, or the person that gives you the test kit say or do to make you more likely to complete it?

- Explain procedure if needed: “This test requires a series of stool samples to be taken and sent in for testing. There are usually 3 cards that three different stool samples are smeared on and sent to the doctor for analysis. FIT needs to be conducted once a year.”

10. What about things that other people could say or do to convince you to complete this test? People like your friends, pastor, co-workers, someone in your family?

11. Once you turn in the test, what would be the best way to learn about the results?

12. What other information would you like to receive with the results?

*At this point, the facilitator should ask if the note taker has any questions.*

These are the different types of screening tests available:


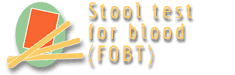
**Stool Test for Blood (FOBT or FIT)**

There are two types of stool test. They are very similar. These tests require a series of stool samples to be taken and sent in for testing. There are usually 3 cards that three different stool samples are smeared on and sent to the doctor for analysis. FOBT or FIT needs to be done once a year.


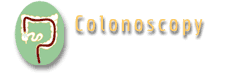
**Colonoscopy**

This test must be conducted either in a hospital or doctors office. A lighted tube is inserted into the rectum and used to view the entire colon. Patients are usually sedated for this test. Colonoscopy needs to be conducted once every 10 years.


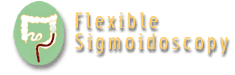
**Flexible Sigmoidoscopy**

This test must be conducted either in a hospital or doctors office. A lighted tube is inserted into the rectum and used to view the lower part of the colon. Patients are usually awake for this test. Sigmoidoscopy needs to be conducted once every 5 years.


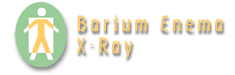
**Barium Enema and X-Ray**

This test must be conducted either in a hospital or doctors office. An X-ray is used to view the colon after a chalky material is inserted for viewing. Barium Enema needs to be conducted once every 10 years.


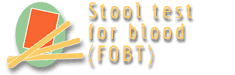

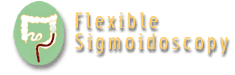
**FOBT/FIT and Flexible Sigmoidoscopy**

This option combines FOBT every year with sigmoidoscopy every 5 years.

This overview of screening options was developed by a research team led by Michael Pignone at the University of North Carolina at Chapel Hill for the CHOICE2 Study funded by the Foundation for Informed Medical Decision Making.
